# Dog owner mental health is associated with dog behavioural problems, dog care and dog-facilitated social interaction: a prospective cohort study

**DOI:** 10.1038/s41598-023-48731-z

**Published:** 2023-12-08

**Authors:** Ana Maria Barcelos, Niko Kargas, Phil Assheton, John Maltby, Sophie Hall, Daniel S. Mills

**Affiliations:** 1 Department of Life Sciences, University of Lincoln, Lincoln, UK; 2 School of Psychology, University of Lincoln, Lincoln, UK; 3 Company Statsadvice.com, Berlin, Germany; 4 School of Psychology and Vision Sciences, University of Leicester, Leicester, UK; 5 School of Medicine, University of Nottingham, NG7 2RD, Nottingham, UK; 6 Department of Life Sciences, University of Lincoln, Lincoln, UK

**Keywords:** Quality of life, Psychology and behaviour, Psychology, Anxiety, Depression, Animal behaviour

## Abstract

Despite numerous qualitative and cross-sectional studies investigating how dog-related factors may impact owners’ well-being, empirical studies to test these causal effects are lacking. This prospective cohort study examined the correlation and potential causal effect of 17 dog-related factors with six well-being outcomes (depression, anxiety, loneliness, suicidal ideation, hedonic well-being and eudaimonic well-being) in dog owners. Over a four-week period, 709 adult dog owners reported their weekly well-being and occurrence of each dog-related factor (e.g. how many times they ran with their dogs). A random intercept cross-lagged panel model (RI-CLPM) with significance threshold set at 0.001 was used. Six factors correlated with poorer owner well-being (i.e. aggressive dog behaviour, fearful dog behaviour, poor dog health, failure to provide for the dog, lack of control over the dog, and dog presence). Only ‘friendly conversation with others due to the dog’ correlated with better well-being. Purposeful reductions in the frequency of dog behavioural and health-related issues are likely to improve owner well-being, as well as greater consistency in dog care (i.e. provide for the dog) and more engagement in friendly dog-facilitated social interactions. No potential causal effects were significant. Further studies investigating causal relationships are essential to improve people’s well-being through dog ownership.

## Introduction

The positive impacts of pet ownership on owner well-being are well documented in the literature^1, 2^ and widely reported by media outlets^3^. However, recent research also suggests several potential factors that could have negative effects on human well-being^4–8^ and animal welfare^9, 10^. For example, it has been reported that pet behavioural problems might lead to poorer mental health in pet owners^4, 7, 8, 11^ and abandonment of pets^9, 10, 12^. According to the social exchange theory, a relationship, such as that between pet and owner, is only maintained if the benefits outweigh the costs^13^. Although it is hard to quantify the benefits and costs, a greater understanding of how pet-owner interactions affect owners’ well-being may permit purposeful improvement not only in the relationship per se but also in owner mental health. For example, to help decrease owner anxiety, interactions or events within the relationship that trigger anxiety could be purposefully minimised and those that reduce anxiety encouraged. Interactions/events related to pets are often referred to as pet-related activities^4, 6^, but in this paper they will be referred to as “pet-related factors”, which is a broader term more suitable for the myriad of circumstances, situations, events in the life of a pet owner.

A few previous studies have investigated associations between specific dog-related factors and owner well-being, such as the impact of dog behavioural problems or dog walking on owner well-being. However, these studies are rarely comprehensive (i.e. various pet-related factors and well-being outcomes), rigorous or large in terms of sample size. For example, in a small cross-sectional study (*n* = 66) dog owners who rated their dogs as more disobedient were more stressed, and owners with less friendly dogs were more depressed and anxious^14^, which could indicate that behavioural problems cause negative changes on owner well-being. In contrast, some studies using an experience sampling design found links between the presence of companion animals and better owner well-being (*n* = 33^15^, *n* = 55^16^, *n* = 159^17^), suggesting that physical proximity to one’s pet could have short term psychological benefits, particularly stress buffering. Therefore, despite the relevance of these studies, an empirical but comprehensive approach that takes into account the myriad of dog-related factors within pet ownership^4, 5^ is still missing.

Another issue is the inconsistencies found in the literature in relation to the impact of dog-related factors on owner well-being. For instance, dog walking is a pet-related factor frequently claimed to be beneficial^18, 19^. However, results from quantitative studies assessing whether owners who walk their dogs more have better well-being (e.g. are less stressed, less depressed) have not found statistically significant relationships (e.g. Ref.^20^, *n* = 477^21^, *n* = 122). Nevertheless, this research field is still in its infancy, as there are various dog-related factors barely explored in relation to owners’ well-being. For instance, one that is gaining increasing scientific attention is dog training. A recent pre-post study with 42 dog owners found that, immediately after a 10-min online-guided training session with their own dogs, owners felt less anxious, and their moods and self-efficacy improved in comparison to before the intervention^22^. There is still much to be explored about the effects of dog-related factors on human well-being, particularly with more representative sample sizes and rigorous designs (e.g. randomised controlled trials, cohort studies^23^). In addition, some dog-related factors have not yet been explored quantitatively in this field of research, and deserve further attention. For example, given the potential therapeutic role of touch on anxiety^24^, are owners who touch their dogs frequently less anxious than others? Or given the avoidance tendencies associated with fear, is the frequency of fearful dog behaviour correlated with loneliness in owners?

Through interviews and focus groups with pet owners, Barcelos et al.^4, 5^, Corrêa et al.^25^ and Ravenscroft et al.^26^ developed a consistent framework^27^, which illustrates how pet-related factors in the lives of pet owners may affect owners’ well-being, both positively and negatively. For example, aggressive dog behaviour was consistently reported to reduce dog owners' hedonic and eudaimonic well-being. The framework lays the groundwork for investigations of high scientific rigour (e.g. randomised controlled trials, cohort studies). In the current study, 17 key pet-related factors (see Table 1 in “Material and methods”) based on the framework described were assessed in regards to their relationship to six well-being outcomes (i.e. depression, anxiety, loneliness, suicidal ideation, hedonic well-being, eudaimonic well-being) in a four-week prospective cohort design. These mental health outcomes are common issues^28^ and the well-being outcomes were chosen due to their relevance to dog owners in previous investigations by the authors^4, 5, 25^. Hedonic well-being is someone’s affect (emotion and mood) and life satisfaction^29^, and eudaimonic well-being represents one’s areas of functioning in life (e.g. self-acceptance, purpose in life)^30^. This methodology (i.e. prospective cohort) allowed for the first time the testing of correlations and some potential causal relationships between specific dog-related factors and aspects of human well-being, e.g. are owners who pet their dogs very often generally less anxious than owners who rarely pet their dogs? Does an increment in the frequency of dog training in a particular week lead to lower depression in the following week?

The aim of this study was to explore the relationship between dog-related factors and owners’ well-being over multiple sample periods during a four-week period. It was hypothesised that the factors previously reported as predominantly positive for human well-being (e.g. tactile interactions, social interactions due to the dog) in the framework would be linked with and lead to better well-being in owners (e.g. less depression), whereas factors seen as predominantly negative (e.g. aggressive dog behaviour, dog poor physical health) would be linked with and lead to worse owner well-being (e.g. greater anxiety or possibly even more suicidal ideation).

## Material and methods

### Ethical approval

This study was approved by the ethics committee of the University of Lincoln (reference number: 2022_0503) and was carried out in accordance with the BPS Code of Ethics and Conduct. Online consent was obtained from participants after they read detailed information about the study (i.e. participant information sheet).

### Participants

The inclusion criteria were to currently own one or more dogs (self-reported ownership) and be at least 18 years old. Participants were recruited on a voluntary basis using non-probability convenience sampling via social media networks (Reddit, Facebook, Twitter, WhatsApp) and on the University of Lincoln’s news website. A virtual flyer about the study was disseminated by the researchers, their friends and by various animal groups/charities (see list in the Acknowledgements) on their own profiles and on groups or pages related to dogs, e.g. dog owners in the UK. In the flyer, there were brief details about the study (i.e. aim of the study and what participants would do), who could participate (i.e. dog owners over 18 years old) and how to access the study (i.e. link to online questionnaires). A prize draw for online shopping vouchers was offered as an incentive to participate. A minimum of 633 participants was sought, based on an a priori sample size calculation (further detail in Statistical analysis).

### Data collection procedure

Participation in the study was anonymous through weekly online questionnaires (QualtricsXM). The data were collected in 2022. After enrolment, in the first week, participants were asked to provide demographic information relating to participants’ age, country, gender, relationship status, whether they were experiencing a physical health condition, whether they were experiencing a mental health condition, how many dogs they had (i.e. one or multiple) and how psychologically close to their dog they felt in a visual scale scored from 0 to 7 (distant from dog to very close to dog), as per Barcelos et al.^6^.

After collection of basic demographics, participants were asked to answer four identical questionnaires online, with a one-week interval between them. In each weekly questionnaire, they reported their own involvement in 17 dog-related factors (Table 1) in the last seven days and rated six different aspects of their well-being (i.e. depression, anxiety, loneliness, eudaimonic well-being, hedonic well-being, and suicidal ideation) over the last seven days. Due to the sensitivity of some of these items, a list of mental health support services was provided to all participants on the first page of the online questionnaires.

**Table 1:** Seventeen dog-related factors assessed in the questionnaires and options for their occurrence/rating in the last 7 days.

Factor	Response options
1. Physical health of your dog (e.g., sickness, injuries experienced)	Excellent (7), very good (6), good (5), neither good nor poor (4), poor (3), very poor (2), extremely poor (1)
2. Running or jogging with your dog	Zero (0), once (1), […], 16 or more (16)
3. Walking your dog	Zero (0), once (1), […], 35 or more (35)
4. Failing to do something for your dog that you should have done (e.g. not able to walk the dog)	Zero (0), once (1), […], 35 or more (35)
5. Dog greeting you at the door	Zero (0), once (1), […], 35 or more (35)
6. Dog presence—proportion of time awake spent in close proximity to your dog	0% to 100% (slider)
7. Friendly conversation with others due to your dog presence	Zero (0), once (1), […], 35 or more (35)
8. Problematic interactions with others related to your dog's presence	Zero (0), once (1), […], 35 or more (35)
9. Dog to human touching—affectionate physical contact initiated by your dog	Less than once a day (0), once a day (1), […], 30 times a day or more (30)
10. Human to dog touching—affectionate physical contact initiated by you	Less than once a day (0), once a day (1), […], 30 times a day or more (30)
11. Aggressive behaviour (e.g. growling, trying to bite, lunging, offensive bark) shown by your dog	Zero (0), once (1), […], 35 or more (35)
12. Fearful/anxious behaviour shown by your dog (e.g. fear of noises, of other individuals, separation anxiety)	Zero (0), once (1), […], 35 or more (35)
13. Barking episodes that caught your attention, e.g. very loud, concerning, unexpected	Zero (0), once (1), […], 35 or more (35)
14. Destroying/chewing/stealing unsuitable items	Zero (0), once (1), […], 35 or more (35)
15. Lack of control over your dog, e.g. poor recall, dog pulls on the lead, runs away	Zero (0), once (1), […], 35 or more (35)
16. House soiling by your dog with urine or faeces	Zero (0), once (1), […], 35 or more (35)
17. Training your dog (e.g. obedience, agility)	Zero (0), once (1), […], 35 or more (35)

### Weekly questions

The 17 dog-related factors (Table 1) chosen for this study were selected due to their relevance in previous investigations^4–6, 25^. Participants self-rated the occurrence of most of these factors over the last seven days, e.g. in the last seven days, how many times have you walked your dog? The only two exceptions were ‘physical health’ of the dog, which was rated on how good/bad it had been in the last seven days, and ‘dog presence’, which was measured by the percentage of awake time the owner had spent with the dog (Table 1).

Depression and anxiety were assessed using the English version of the 9-item Patient Health Questionnaire (PHQ-9^31^) and the 7-item Generalised Anxiety Disorder scale (GAD-7^32^), respectively. Participants were asked to indicate how often (not at all, several days, more than half the days, nearly every day) they were bothered by particular situations over the last seven days, e.g. ‘little interest or pleasure in doing things’ (from the PHQ-9 scale), ‘feeling nervous, anxious, or on edge’ (from the GAD-7 scale). Scores can range from 0 to 27 and 0 to 21 on the depression and anxiety scale, respectively. Cronbach’s Alpha (α) for the PHQ-9 scale on weeks one to four was α = 0.881, 0.879, 0.892, and 0.895; and for the GAD-7 scale was α = 0.896, 0.900, 0.907, and 0.913. All these values are within reported acceptable α ranges (i.e. 0.70 to 0.95^33^).

Loneliness was measured with the English version of the validated 3-item UCLA Loneliness Scale^34^. Besides being a short instrument, it is internationally used and is recommended by the Office for National Statistics^35^. Accordingly, participants were asked to think about their last seven days and indicate how often (hardly ever or never, some of the time, often) they felt that they lacked companionship (1), they felt left out (2), and they felt isolated from others (3). Scores can range from 3 to 9. The loneliness scale had good internal consistency from week one to four: α = 0.859, 0.868, 0.899, 0.893.

To assess hedonic and eudaimonic well-being in the last seven days the English version of the Mental Health Continuum-Short Form^36^ was used. Three items on this scale measure hedonic well-being (e.g. feeling happy) and six items assess eudaimonic well-being (e.g. feeling that you like most parts of your personality). These last six items cover the main six elements of eudaimonic well-being as per Ryff^30^: autonomy, purpose in life, environmental mastery, positive relations with others, personal growth and self-acceptance. The three items of hedonic well-being cover both affect (i.e. mood, emotions) and life satisfaction, in accordance with Diener et al.^29^. Participants could choose the following options: every day, almost every day, about 2 or 3 times a week, about once a week, or never. The option ‘once or twice a month’ from the original scale was not needed, because we assessed participants’ well-being in the last 7 days, rather than in the last month. Hedonic well-being scores could range from 3 to 15, and eudaimonic well-being scores from 6 to 30. Cronbach’s Alpha for the scale of hedonic well-being was 0.858, 0.884, 0.912, 0.907 on weeks one to four; and for eudaimonic well-being was 0.816, 0.842, 0.872, 0.883.

Finally, suicidal ideation was also assessed in each of the questionnaires. The question “in the last 7 days, have you thought of taking your life, even if you would not really do it?” was chosen for this assessment due to its simplicity and usage in the Adult Psychiatric Morbidity Survey in England^37^ and in a study about mental health in the veterinary profession^38^. The following response options were given: yes, no, I prefer not to say.

### Statistical analysis

Based on a simulation with small factor vs well-being effects (numerically solved to have standardised coefficients of 0.1), set against moderate correlations and autocorrelations, and using a fixed effects estimator (controlling for participant means as a fixed effect) as a more computationally efficient proxy for random intercept cross-lagged panel model (RI-CLPM), a sample size of 633 was estimated to give 80% power at α = 0.001.

To assess the relationship between the 17 dog-related factors predicted changes in owners’ well-being (i.e. depression, anxiety, loneliness, eudaimonic and hedonic well-being), RI-CLPM was used^39^. This allows the assessment of both stable and dynamic relationships between independent variables and a given outcome measure. Before fitting the model, well-being scores were standardised (subtraction of mean and division by standard deviation), and factor occurrences were log_2_ transformed, with 0 replaced with log_2_(0.5). By doing this, regression coefficients were the number of standard deviations (SDs) moved by the well-being score in the presence of a doubling of the factor in question (or a jump from 0 times per week to 1 time per week). The log transform also removed a considerable amount of the skewness in the factor variables. Nonetheless, a few variables were still noticeably non-normal. In the case of structural equation models with continuous, non-normal data, a common recommendation, e.g. Kline ^40^, is to estimate the model using maximum likelihood, but to use robust standard errors and test statistics that account for the kurtosis in the distribution^41^. The only factor that was not log transformed was the “physical health of your dog”, which was recorded on a seven-point Likert-scale, which would not make sense under a log transformation. This variable was left untransformed and treated as if continuous: coefficients from regressing onto this variable represent the number of SDs moved by the mood variable, when the physical condition of the dog moves by one point on the seven-point scale.

The RI-CLPM model was fitted using the lavaan package in R (version 4.1.0) based on the structural equation modeling (SEM) syntax of Mulder and Hamaker^42^. The RI-CLPM decomposed the correlation structure of the data into a number of components: first, 'between-person' correlations of participants' four-week average scores (in both factors and well-being) are estimated to identify general associations between factors' occurrences and well-being outcomes, e.g. whether people who walked their dogs more often during the four weeks were less depressed in this period. Second, 'within person' residuals are calculated from week-to-week variations in the occurrences of factors and well-being outcomes. The model is cross-lagged, which is to say that the within-person residuals of a given score are regressed onto the within-person well-being and factor residuals from the previous week and vice versa. This attempts to identify causality, on the principle that something that occurred in the previous week cannot be “caused” by what is happening this week. In effect, these regressions attempt to identify causality by asking the question "does a factor (e.g. walking) last week impact on well-being (e.g. depression) in the following week?" and, conversely, “does well-being last week impact on this week’s factor?”. Needless to say, causal relationships may not necessarily manifest in this way. Finally, any unexplained variance in the within-person residuals are further correlated, which asks the question “do weeks with more dog-related factor tend to correlate with a change in well-being?”.

Contrary to the other well-being outcomes, suicidal ideation was categorically assessed (yes, no, prefer not to say), thus, a separate RI-CLPM model was fitted for this variable and each of the 17 dog-related factors. The principles and methods described above also apply here, except that the models were fit using diagonally weighted least squares (a method that treats the binary yes/no values as observations of a normally distributed latent variable).

As this study assessed numerous well-being outcomes and dog-related factors in the RI-CLPM models, a more conservative statistical significance threshold of p < 0.001 (***) has been used. This is around twice the alpha of a Bonferroni adjustment applied to each grid.

## Results

### Demographics

Out of 1133 initial respondents who answered the first questionnaire, 709 of them finished the study (i.e. four weekly surveys), which represents a completion rate of 62.6%. Participant age ranged from 18 to 84 years old. Most were female (n = 647, 91.3%), lived in the United Kingdom (n = 436, 61.5%) and were in a relationship (n = 471, 66.4%). Around one-quarter of the participants reported that they experienced a mental health (n = 178, 25.1%) or physical (n = 195, 27.5%) condition. A little more than half of participants owned only one dog (n = 415, 58.5%) and the majority perceived themselves as moderately to highly close to their dogs—664 dog owners (93.7%) scored from 4 to 7 on the visual scale of psychological closeness to the dog. Further detail about participants' characteristics is available in the Supplementary material (Table S1).

### Average well-being and occurrence of factors

The mean and median for the occurrence of each dog-related factor and well-being scores per week over the four weeks are provided in Table 2. In addition, within-person and between-person standard deviation of the occurrence of factors and well-being scores are available in the Supplementary material (Table S2). Factors show a broad spread in their frequencies making prediction of variation a realistic goal.

**Table 2:** The average and median values for the weekly occurrence of the 17 dog-related factors and for participants’ weekly depression, anxiety loneliness, eudaimonic and hedonic well-being scores (n = 709).

	Mean	SD	Median	Min	Max	IQ1	IQ3
**Dog-related factor**							
Physical health of dog (1 = extremely poor, 7 = extremely good)	5.91	1.30	6	1	7	5	7
Running/jogging with dog	0.83	2.37	0	0	16	0	0
Walking dog	10.10	6.68	9	0	35	5	14
Fail to do something for dog	1.94	3.09	1	0	35	0	3
Dog greeting at the door	9.55	7.34	7	0	35	5	14
Dog presence (% of time together)	73.05	19.60	78	0	100	62	90
Friendly conversation with others due to dog presence	5.54	5.93	4	0	35	2	7
Problematic interaction with others while out with dog	1.16	2.95	0	0	35	0	1
Dog to human touching	8.41	7.71	5	0	30	3	10
Human to dog touching	8.60	7.52	6	0	30	4	10
Aggressive dog behaviour	1.31	3.08	0	0	35	0	1
Fearful dog behaviour	2.49	3.90	1	0	35	0	3
Distinct barking episodes	3.47	5.70	2	0	35	0	4
Destroy/chew/steal	0.74	2.47	0	0	35	0	0
Lack of control over dog	1.44	3.18	0	0	35	0	2
House soiling	0.37	1.71	0	0	35	0	0
Training dog	5.69	6.58	4	0	35	1	7
**Well-being outcome**							
Depression (PHQ-9)	5.46	5.38	4	0	27	2	8
Anxiety (GAD-7)	4.56	4.78	3	0	21	1	6
Loneliness	4.27	1.70	3	3	9	3	5
Eudaimonic well-being	16.79	5.07	18	0	24	14	21
Hedonic well-being	9.09	2.63	9	0	12	8	11

Suicidal ideation in the previous seven days occurred on average in about 10% of participants (9.63%). The large majority (88.61%) did not have suicidal thoughts and 1.76% of participants preferred not to say anything about this item.

### Model fit

The RI-CLPM testing the impact of the 17 dog-related factors on the five well-being outcomes (i.e. depression, anxiety, loneliness, eudaimonic and hedonic well-being) showed excellent measures of model fit, with an RMSEA_robust_ near zero (0.021, 95% CI 0.018, 0.023, p = 1), and CFI_robust_ close to one (0.981). RMSEA stands for root mean square error of approximation and CFI for comparative fit index, which are common measures of model fit. Hu and Bentler^43^ recommend that RMSEA be below 0.06 and CFI above 0.95. The model-fit Satorra-Bentler scaled Chi-square was statistically significant (χ^2^(2739) = 3456.46, *p* < 0.001), which means that the null hypothesis of perfect model fit was rejected by this test. However, given the large sample size, tiny deviations from perfection make this inevitable; given the excellent measures of fit, this result is not considered a cause for concern.

The set of RI-CLPMs assessing how each of the 17 dog-related factors may affect suicidal ideation were not as good: while all CFIs were at 0.95 or above, around a third of them showed RMSEA > 0.06, with the max RMSEA being 0.094, and with almost all (> 90%) with RMSEA < 0.08. This is only slightly above the desirable range but may well mean that the model used for these variables is over-simplistic and unable to fully reconstruct the correlation matrix of the data. On account of the relative crudeness of these models, as well as their poorer model fit statistics, results for the suicidal ideation variable should be interpreted with caution.

### Dog-related factors correlated with well-being outcomes

The between-person correlations of participants’ four-week average scores in both factors and well-being are given in Table 3. Owners who more often failed to do something for their dog (e.g. not taking the dog for a walk) reported poorer (*p* < 0.001) scores in all aspects of well-being assessed. Three other factors associated with owners with lower well-being were identified: aggressive dog behaviour, fearful dog behaviour, and lack of control over the dog. Owners of dogs who reported aggression more often felt lonelier, depressed, anxious, and had lower hedonic well-being than their counterparts. Similarly, owners of dogs more often fearful were more depressed and anxious, and owners who lacked control over their dogs (e.g. dog pulling on the lead, poor recall response) more frequently were more likely to have suicidal thoughts in the last seven days.

**Table 3:** Between-person correlations of dog owners’ average scores over the four-week period (n = 709).

Dog-related factor	Depression (PHQ-9)	Anxiety (GAD-7)	Loneliness (UCLA)	Eudaimonic well-being	Hedonic well-being	Suicidal ideation
Physical health of dog	− 0.16**	− 0.12*	− 0.01	0.12**	0.14**	0.02
Running/jogging with dog	− 0.06	0.04	− 0.06	0.08*	0.07*	0.07
Walking dog	− 0.14**	− 0.12**	− 0.08	0.15**	0.14**	− 0.04
Fail to do something for dog	0.39***	0.34***	0.21***	− 0.30***	− 0.31***	0.16***
Dog greeting at the door	− 0.09	− 0.07	− 0.11*	0.15**	0.14**	− 0.02
Dog presence	0.11*	0.04	0.02	− 0.05	− 0.04	0.17***
Friendly conversation with others due to dog presence	− 0.17***	− 0.15***	− 0.12**	0.27***	0.27***	− 0.01
Problematic interaction with others while out with dog	0.13*	0.15*	0.14*	0.05	0.01	0.06
Dog to human touching	0.11*	0.13**	0.04	0.02	0.02	0.10**
Human to dog touching	0.08	0.11*	0.07	0.02	0.02	0.10*
Aggressive dog behaviour	0.23***	0.19***	0.19***	− 0.08	− 0.16***	0.11*
Fearful dog behaviour	0.18***	0.20***	0.09	− 0.02	− 0.07	0.09*
Distinct barking episodes	0.09*	0.08	0.04	− 0.04	− 0.03	0.11**
Destroy/chew/steal	0.04	0.03	0.06	0.02	0.00	0.04
Lack of control over dog	0.12*	0.11*	0.11*	− 0.06	− 0.05	0.13***
House soiling	0.06	0.04	0.02	− 0.03	− 0.02	− 0.02
Training dog	0.09*	0.11*	− 0.00	0.06	0.03	0.09*

Values are Pearson correlation coefficients obtained from the RI-CLPM. * < 0.05, ** < 0.01, *** < 0.001. The measurement used to assess each factor is available in Table 1.

The only potentially beneficial factor identified in these correlations was having friendly conversations with others due to the dog (Table 3). Owners who engaged more often in these social interactions reported less depression, less anxiety and better hedonic and eudaimonic well-being.

In contrast, there was a positive correlation between dog presence and suicidal ideation. Owners who reported thinking about suicide more often spent more time with their dogs.

The within-person correlations (Table 4) indicate whether higher occurrence of an activity factor in a particular week is correlated with higher/lower well-being scores *in that week.* The physical health of the dog appears to be closely linked to owners’ feelings (Table 4). When the dogs were in poorer health condition, owners reported more symptoms of depression and anxiety, whereas better physical health of the dog was positively correlated with better hedonic well-being. Similar to the between-person correlations, greater occurrence of failing to do something for the dog, fearful dog behaviour and lack of control over the dog were correlated with worse well-being outcomes (Table 4). Failing more often to do something for the dog was linked with more depression and lower hedonic well-being; more episodes of fearful dog behaviour were correlated with worse eudaimonic well-being; and lacking control over the dog more often was positively associated with anxiety and depression.

**Table 4:** Within-person correlations of dog owners’ average scores over the four-week period (n = 709).

Dog-related factor	Depression (PHQ-9)	Anxiety (GAD-7)	Loneliness (UCLA)	Eudaimonic well-being	Hedonic well-being	Suicidal ideation
Physical health of dog	− 0.17***	− 0.16***	− 0.06*	0.09**	0.14***	− 0.06
Running/jogging with dog	0.02	− 0.02	0.01	0.03	0.02	− 0.03
Walking dog	− 0.02	− 0.03	− 0.02	0.03	0.03	0.05
Fail to do something for dog	0.13***	0.10**	0.08**	− 0.06	− 0.13***	− 0.01
Dog greeting at the door	0.01	0.03	− 0.01	− 0.03	0.01	− 0.00
Dog presence	− 0.01	0.00	0.02	− 0.02	0.00	− 0.05
Friendly conversation with others due to dog presence	− 0.01	0.04	− 0.04	0.07*	0.02	0.08
Problematic interaction with others while out with dog	0.05	0.03	0.03	0.02	− 0.02	− 0.01
Dog to human touching	0.01	0.00	0.02	0.04	0.02	0.00
Human to dog touching	0.05	0.05	0.04	− 0.01	− 0.04	− 0.02
Aggressive dog behaviour	0.06	0.05	0.01	− 0.02	0.01	− 0.00
Fearful dog behaviour	0.11**	0.11**	0.07*	− 0.11***	− 0.07*	0.03
Distinct barking episodes	0.03	0.05	0.01	0.00	− 0.02	− 0.00
Destroy/chew/steal	0.08**	0.11**	0.01	− 0.06	− 0.03	− 0.02
Lack of control over dog	0.14***	0.16***	0.07	− 0.11**	− 0.09**	0.03
House soiling	0.06	0.07	0.02	− 0.04	− 0.04	− 0.03
Training dog	− 0.04	− 0.05	− 0.03	0.00	0.02	0.01

Values are Pearson correlation coefficients obtained from the RI-CLPM. * < 0.05, ** < 0.01, *** < 0.001. The measurement used to assess each factor is available in Table 1.

The 95% confidence intervals of the correlation coefficients in Tables 3 and 4 are available in the Supplementary Material (Tables S3 and S4).

### Lack of causal effect identified between factors and well-being outcomes

The cross-lagged coefficients of the RI-CLPM (Table S5, Supplementary material) represent one week’s well-being regressed on last week’s factor, and so illustrate how a change in factor last week precedes a change in well-being in the current week. None of the cross-lagged coefficients was significant, indicating there was no evidence of this type of causal association.

Likewise, the cross-lagged coefficients for the current week's factor regressed on last week's well-being (Supplementary material, Table S6) also indicated that there was no evidence that changes in well-being outcomes might cause changes in the occurrence of dog-related factors in the following week.

## Discussion

To assess the correlation and potential causal relationship between 17 dog-related factors and owners' well-being (i.e. depression, anxiety, loneliness, hedonic well-being, eudaimonic well-being, suicidal ideation), we evaluated the weekly factor and well-being reports of 709 dog owners, over a four-week period. Our results show that having more 'friendly conversations with others due to dog presence' is correlated with some aspects of better owner well-being (e.g. lower depression and anxiety, higher eudaimonic and hedonic well-being). In contrast, higher occurrences of aggressive dog behaviour, fearful dog behaviour, poor dog health, failure to provide for the dog, lack of control over the dog, and dog presence were correlated with poorer owner well-being outcomes. However, further tests to potentially detect the causal nature of this relationship were not statistically significant.

Factors related to dog behavioural problems—dog aggressive behaviour, fearful/anxious behaviour, and lack of control over the dog (e.g. dog pulling on the lead, poor recall^44, 45^)—were correlated with poorer owner well-being. Previous investigations also have found that owners who perceive their dogs' behaviour as more problematic have poorer mental health^14^, are less satisfied with their dogs and have a greater intent to abandon them^12^. Indeed, behavioural problems are one of the main causes of animal relinquishment^9, 10^. Therefore, behavioural issues may indicate not only poor dog welfare but also poor owner well-being which is in line with owners' own perceptions^4–7, 25^. This could be caused by repeated stress triggered by the behavioural issue per se or by a sense of responsibility for the behaviour of the dog. Owner mental health problems may also feedback negatively on their dogs’ behaviour, as dogs can mirror their owners' emotions (e.g. Sundman et al.^46^) and can be “victims” of their owners’ symptoms (e.g. lower motivation to accomplish daily tasks, social isolation^47^). For instance, these symptoms might lead to poorer care-taking or reduced exposure of the dog to social stimuli and, as a consequence, an increase in behavioural problems. Thus, bidirectional effects between dog behavioural problems and owner mental health issues are strongly suggested as an explanation for the study results.

We found that owners who were more likely to think about suicide spent a greater proportion of their awake time with their dogs. Three studies using experience sampling design^48^ have found that the presence of companion animals is associated with better well-being in owners^15–17^ and dog owners’ reports indicate that dog presence is predominately beneficial for their well-being, e.g. decreasing negative feelings^4–6, 25^. Furthermore, based on interviews with 71 pet owners who had thought about or attempted suicide, Love^11^ suggested that the company of pets is a protective factor against suicide. These findings raise the possibility that dog owners with more suicidal thoughts seek the company of their dogs more often to alleviate their negative affect (e.g. stress, anxiety, sadness). It might be that spending more time with the dog in this study reflects something like greater social isolation, which is one of the main risk factors for suicide-related outcomes (e.g. suicidal ideation and suicide^49^). In this context, dogs might be one of the sole sources of social support and potentially a protective factor against suicide, such as suggested by Love^11^ and Barcelos et al.^5^.

Failing more often to do something for the dog (e.g. not able to walk or feed the dog), was correlated with poorer owner well-being in all aspects assessed. The sense of failure to provide optimal care for the dog per se is an obvious explanation for poorer well-being, as it may contribute to guilt and bad feelings about the relationship with the dog^27, 50^. It is also possible that factors such as repeated failure reflect a disturbed owner routine which counteracts the benefits to human well-being suggested by having a dog-related routine^4, 5, 25, 51, 52^. Barcelos et al.^27^ discuss in detail the well-being benefits and challenges of having a pet-related routine and caring for a pet. Anhedonia (i.e. lower motivation to accomplish daily tasks, lack of interest or motivation to engage in daily activities), a symptom of several mental health issues (e.g. depression^53^), or other routine disturbances caused by the owner's well-being issues, might also increase the risk of failures in dog caretaking duties.

Another important finding concerns the correlations between poorer dog physical health and increased depression and anxiety in owners. Dealing with poor health and the end of dogs’ lives has been reported by dog owners as one of the most challenging dog-related factors (e.g. Ref.^4–6, 54^). In a cross-sectional study with 238 pet owners, those who owned a pet with a chronic or terminal disease were more anxious, depressed, stressed, and had lower quality of life than owners with healthy animals^55^. Therefore, we suggest that physical health problems in dogs could exacerbate or increase the risk of symptoms of depression and anxiety in owners. The inverse relationship is also plausible, as mental health issues might interfere with the owners' ability to contribute to their dog's optimal physical health.

The only factor correlated with more positive well-being outcomes was 'friendly conversations with others due to dog presence'. Owners who engaged in these conversations more often were typically less anxious, less depressed and had greater eudaimonic well-being. Considering previous studies indicating that the social lubricant effect of dogs not only increases social interactions^56^ but also improves one's sense of social support^57^, it is plausible that engaging in more conversations with others due to the dog indeed helps minimise symptoms of depression and anxiety, and is boosting owners' eudaimonic well-being (e.g. positive relations with others, self-acceptance). However, it is also likely that social withdrawal, which features in many mental health issues^58^, is responsible for the lower frequency of dog-related social interactions in these individuals. Thus, a bidirectional relationship is suggested.

One limitation of our results is that the causal relationship could not be determined, given the lack of significance in the cross-lagged coefficients. Importantly, a nonsignificant test result (p > 0.001 herein) does not mean that there is no causal association between factors and human well-being^59^, only that we could not detect it. In a longer study, greater than four weeks, a causal relationship may be found. If we had used p < 0.05 instead of p < 0.001 as the significance threshold, five dog-related factors (e.g. dog walking, dog training) would be significant in these tests of cause-effect (Table S5 in the Supplementary material). However, the more rigorous threshold (p < 0.001) reduces the chance of identifying random factors as important for well-being (type II error), when in fact significant findings are simply occurring due to the high number of tests being performed.

Given our previous qualitative work^4–6, 25^ we chose to use the frequency of most factors as our predictor of owner well-being, however, it is possible some other metric may be more important for some of these factors (e.g. duration, time-specific regularity). In addition, due to the self-report nature of the study, the data is subjective. For example, it is possible that two dogs with similar physical health status received different ratings as different owners perceive their dogs differently. Likewise fearful behaviour in dogs might be more easily noticed by some owners than others^60^. Thus, the results must be interpreted as a reflection of owners' own perceptions. As such, it is also possible that owners with poorer mental health rated the dog-related factors more negatively, which may contribute to the significant correlations found in the study.

Other limitations are the large proportion of female participants (91.3%), which is common in studies of human-animal interaction^61^, and the dropouts from the beginning to the end of the study, which is a common issue in longitudinal studies^62^. Finally, although human personality was not assessed in this study, it is important to acknowledge that personality and well-being reciprocally influence each other over time^63^. In fact, human personality and dog-related factors are likely to be interlinked, as previous studies have indicated^64, 65^. For example, it is possible that owner personality affects the frequency of aggressive behaviour in the dog or how often owners have friendly conversations with others.

In this study, we assessed how changes in dog-related factors were correlated and/or had a potential causal effect on dog owners' well-being over a four-week period. Based on the correlation results, we suggest that purposeful decrements in the occurrence of dog behavioural and health-related issues are likely to improve owners' mental health, as well as being more consistent in the care of the dog (i.e. 'failing' less). For instance, animal behaviour management (e.g. through an animal behaviourist, dog training classes) and adjustments in the owner's own routine through self-motivation or the support of a professional (e.g. psychotherapist, care worker) might help in achieving these benefits. Engaging more often in friendly social interactions while out with the dog (e.g. during walks or meetings in dog-friendly places) also seems to have an important effect. New empirical studies with different methodologies (e.g. longer duration, RCT) are recommended to better evaluate the cause-effect between dog-related factors and well-being outcomes.

### Supplementary Information

Supplementary Tables.

## Data Availability

Data used for analysis can be accessed on Open Science Framework through this link: https://osf.io/axhsf/?view_only=90d3de65beda4616840da3d5505968d9.
