# Supplementary material for: Dog owner mental health is associated with dog behavioural problems, dog care and dog-facilitated social interaction: a prospective cohort study
Source: Sci Rep. 2023 Dec 8;13:21734. doi: 10.1038/s41598-023-48731-z (PMC10709316; doi:10.1038/s41598-023-48731-z)
Supplement: Supplementary file 1 — Supplementary Tables. [file 41598_2023_48731_MOESM1_ESM.docx]

**Supplementary material**

**Dog owner mental health is associated with dog behavioural problems and**

**dog-related activities in a prospective cohort study**

Ana Maria Barcelos, Niko Kargas, Phil Assheton, John Maltby, Sophie Hall & Daniel S. Mills

**Table S1.** Characteristics of the 709 dog owners who completed the study

| **Value** | **n** | **%** | **Value** | **n** | **%** |
| --- | --- | --- | --- | --- | --- |
| 18-24 | 22 | 3.1% | In a relationship | 471 | 66.4% |
| 25-34 | 163 | 23.0% |  |  |  |
| 35-44 | 130 | 18.3% | Experiencing a physical | 195 | 27.5% |
| 45-54 | 162 | 22.8% | health condition |  |  |
| 55-64 | 147 | 20.7% |  |  |  |
| 65-74 | 66 | 9.3% | Experiencing a mental | 178 | 25.1% |
| 75-84 | 19 | 2.7% | health condition |  |  |
| 85 or more | 0 | 0.0% |  |  |  |
|  |  |  | Own one dog | 415 | 58.5% |
| Female | 647 | 91.3% | Own more than one dog | 294 | 41.5% |
| Male | 47 | 6.6% |  |  |  |
| Non-binary | 12 | 1.7% | 1. No overlap - distant from dog | 4 | 0.6% |
| Other | 1 | 0.1% | 2 | 16 | 2.3% |
| Prefer not to say | 2 | 0.3% | 3 | 25 | 3.5% |
|  |  |  | 4 | 107 | 15.1% |
| United Kingdom | 436 | 61.5% | 5 | 170 | 24.0% |
| United States | 118 | 16.6% | 6 | 189 | 26.7% |
| Canada | 24 | 3.4% | 7. A lot of overlap - | 198 | 27.9% |
| Germany | 16 | 2.3% | very close to dog |  |  |
| Australia | 16 | 2.3% |  |  |  |
| Brazil | 5 | 0.7% |  |  |  |
| Other | 94 | 13.3% |  |  |  |

**Table S2**. Standard deviations between and within participants, as estimated by the model (RI-CLPM).

|  |  | **SD_between_** | |  | **SD_within_** | |
| --- | --- | --- | --- | --- | --- | --- |
|  |  | **SD** | **2^2xSD^** |  | **SD** | **2^2xSD^** |
| Physical health of dog |  | 0.91 |  |  | 0.89 |  |
| Running/jogging with dog |  | 0.86 | 2.78 |  | 0.74 | 2.21 |
| Walking dog |  | 1.09 | 2.64 |  | 0.70 | 2.46 |
| Fail to do something for dog |  | 0.91 | 4.63 |  | 1.10 | 2.66 |
| Dog greeting at the door |  | 1.17 | 2.75 |  | 0.73 | 2.63 |
| Dog presence |  | 0.34 | 1.89 |  | 0.46 | 1.51 |
| Friendly conversation with others due to dog presence |  | 1.21 | 3.85 |  | 0.97 | 2.94 |
| Problematic interaction with others while out with dog |  | 0.68 | 4.11 |  | 1.02 | 2.36 |
| Dog to human touching |  | 1.07 | 2.50 |  | 0.66 | 2.42 |
| Human to dog touching |  | 1.07 | 2.54 |  | 0.67 | 2.42 |
| Aggressive dog behaviour |  | 0.96 | 3.16 |  | 0.83 | 2.47 |
| Fearful dog behaviour |  | 1.03 | 3.62 |  | 0.93 | 2.71 |
| Distinct barking episodes |  | 1.38 | 3.61 |  | 0.93 | 3.26 |
| Destroy/chew/steal |  | 0.84 | 2.24 |  | 0.58 | 2.06 |
| Lack of control over dog |  | 1.00 | 3.12 |  | 0.82 | 2.51 |
| House soiling |  | 0.62 | 1.85 |  | 0.44 | 1.70 |
| Training dog |  | 1.51 | 3.58 |  | 0.92 | 3.46 |
|  |  |  |  |  |  |  |
| Depression (PHQ-9) |  | 0.87 |  |  | 0.46 |  |
| Anxiety (GAD-7) |  | 0.85 |  |  | 0.50 |  |
| Loneliness (UCLA) |  | 0.85 |  |  | 0.51 |  |
| Eudaimonic well-being |  | 0.88 |  |  | 0.45 |  |
| Hedonic well-being |  | 0.87 |  |  | 0.48 |  |
| Suicidal ideation |  | 0.18 |  |  | 0.23 |  |
|  |  |  |  |  |  |  |

Note. Values are on the transformed scale. For mood scales (which were standardised), this means that squares of SDbetween and SDwithin sum up to roughly 1 and that each of these SDs can be interpreted as relative to the overall SD of that score in the broader set of all scores recorded. For log2 transformed activity scores, the extra column 2^2xSD^ gives a more intuitive interpretation of the SD: this number represents the ratio between a relatively high scoring (1SD above average) and relatively low scoring (1SD below average) week. For example, from SDbetween, an owner who trains quite a lot might have on average 3.58 times as many training sessions per week as one who trains relatively little; from SDwithin, in a "good" week, a particular owner might have 3.46 times as many training sessions as the same owner in a "bad" week. Overall, activities show a broad spread in their frequencies making prediction of variation a realistic goal. In addition, for a given person, almost all activities occur two to three times as frequently in a typical high week (one geometric SD above average) vs a typical low week (one geometric SD below average) as well as for a typically high participant vs typically lower participant (Supplementary material).  Only percent (%) awake time with the dog and house soiling show a factor of less than 2 (1.51 and 1.83 for within-person (typical good week vs typical bad week) and between person (typically high participant vs typically low participant) SDs.

**Table S3**. Between-person correlations of dog owners' average scores over the four-week period, including the 95% confidence interval.

| **Dog-related activity** | **Depression (PHQ-9)** | | **Anxiety (GAD-7)** | | **Loneliness (UCLA)** | | **Eudaimonic well-being** | | **Hedonic well-being** | | **Suicidal ideation** | |
| --- | --- | --- | --- | --- | --- | --- | --- | --- | --- | --- | --- | --- |
| Physical health of dog | -.16 | ** | -.12 | * | -.01 |  | .12 | ** | .14 | ** | .02 |  |
|  | [-.26, -.06] | | [-.23, -.02] | | [-.11, .08] | | [0.03, .21] | | [0.05, .23] | | [-.07, .11] | |
| Running/jogging with dog | -.06 |  | .04 |  | -.06 |  | .08 | * | .07 | * | .07 |  |
|  | [-.13, .02] | | [-.05, .13] | | [-.15, .03] | | [0.01, .15] | | [0.00, .14] | | [-.00, .15] | |
| Walking dog | -.14 | ** | -.12 | ** | -.08 |  | .15 | ** | .14 | ** | -.04 |  |
|  | [-.24, -.05] | | [-.21, -.03] | | [-.16, .01] | | [0.06, .24] | | [0.05, .24] | | [-.13, .06] | |
| Fail to do something for dog | .39 | *** | .34 | *** | .21 | *** | -.30 | *** | -.31 | *** | .16 | *** |
|  | [0.30, .48] | | [0.25, .43] | | [0.11, .30] | | [-.39, -.21] | | [-.40, -.21] | | [0.07, .26] | |
| Dog greeting at the door | -.09 |  | -.07 |  | -.11 | * | .15 | ** | .14 | ** | -.02 |  |
|  | [-.18, .01] | | [-.16, .03] | | [-.21, -.02] | | [0.05, .24] | | [0.04, .23] | | [-.09, .04] | |
| Dog presence | .11 | * | .04 |  | .02 |  | -.05 |  | -.04 |  | .17 | *** |
|  | [0.01, .21] | | [-.07, .14] | | [-.10, .14] | | [-.16, .06] | | [-.15, .08] | | [0.09, .24] | |
| Friendly conversation with others due to dog presence | -.17 | *** | -.15 | *** | -.12 | ** | .27 | *** | .27 | *** | -.01 |  |
|  | [-.26, -.08] | | [-.24, -.06] | | [-.20, -.03] | | [0.20, .35] | | [0.19, .36] | | [-.08, .06] | |
| Problematic interaction with others while out with dog | .13 | * | .15 | * | .14 | * | .05 |  | .01 |  | .06 |  |
|  | [0.01, .25] | | [0.03, .27] | | [0.02, .26] | | [-.05, .16] | | [-.10, .11] | | [-.05, .16] | |
| Dog to human touching | .11 | * | .13 | ** | .04 |  | .02 |  | .02 |  | .10 | ** |
|  | [0.02, .20] | | [0.03, .22] | | [-.05, .13] | | [-.07, .10] | | [-.06, .11] | | [0.03, .18] | |
| Human to dog touching | .08 |  | .11 | * | .07 |  | .02 |  | .02 |  | .10 | * |
|  | [-.01, .17] | | [0.02, .21] | | [-.02, .15] | | [-.07, .10] | | [-.06, .11] | | [0.02, .17] | |
| Aggressive dog behaviour | .23 | *** | .19 | *** | .19 | *** | -.08 |  | -.16 | *** | .11 | * |
|  | [0.14, .32] | | [0.09, .28] | | [0.09, .30] | | [-.18, .01] | | [-.25, -.06] | | [0.02, .20] | |
| Fearful dog behaviour | .18 | *** | .20 | *** | .09 |  | -.02 |  | -.07 |  | .09 | * |
|  | [0.08, .28] | | [0.10, .30] | | [-.01, .19] | | [-.12, .08] | | [-.17, .03] | | [0.00, .18] | |
| Distinct barking episodes | .09 | * | .08 |  | .04 |  | -.04 |  | -.03 |  | .11 | ** |
|  | [0.00, .19] | | [-.01, .17] | | [-.04, .13] | | [-.13, .05] | | [-.12, .07] | | [0.04, .18] | |
| Destroy/chew/steal | .04 |  | .03 |  | .06 |  | .02 |  | .00 |  | .04 |  |
|  | [-.04, .12] | | [-.05, .12] | | [-.03, .15] | | [-.06, .09] | | [-.07, .08] | | [-.02, .10] | |
| Lack of control over dog | .12 | * | .11 | * | .11 | * | -.06 |  | -.05 |  | .13 | *** |
|  | [0.01, .22] | | [0.01, .21] | | [0.02, .21] | | [-.16, .03] | | [-.15, .04] | | [0.05, .21] | |
| House soiling | .06 |  | .04 |  | .02 |  | -.03 |  | -.02 |  | -.02 |  |
|  | [-.02, .14] | | [-.04, .13] | | [-.06, .10] | | [-.11, .06] | | [-.10, .07] | | [-.07, .03] | |
| Training dog | .09 | * | .11 | * | -.00 |  | .06 |  | .03 |  | .09 | * |
|  | [0.00, .17] | | [0.02, .19] | | [-.09, .09] | | [-.03, .14] | | [-.06, .11] | | [0.01, .17] | |

Note. Values are Pearson correlation coefficients and 95% confidence intervals.

*<.05, **<.01, ***<.001; p<0.001 = significance threshold for interpretation

**Table S4** Within-person correlations of dog owners' average scores over the four-week period, including the 95% confidence interval.

| **Dog-related activity** | **Depression**  **(PHQ-9)** | | **Anxiety (GAD-7)** | | **Loneliness (UCLA)** | | **Eudaimonic well-being** | | **Hedonic well-being** | | **Suicidal ideation** | |
| --- | --- | --- | --- | --- | --- | --- | --- | --- | --- | --- | --- | --- |
| Physical health of dog | -.17 | *** | -.16 | *** | -.06 | * | .09 | ** | .14 | *** | -.06 |  |
|  | [-.23, -.10] | | [-.23, -.09] | | [-.11, -.01] | | [0.02, .15] | | [0.07, .21] | | [-.16, .04] | |
| Running/jogging with dog | .02 |  | -.02 |  | .01 |  | .03 |  | .02 |  | -.03 |  |
|  | [-.05, .08] | | [-.09, .05] | | [-.05, .06] | | [-.03, .10] | | [-.04, .09] | | [-.14, .08] | |
| Walking dog | -.02 |  | -.03 |  | -.02 |  | .03 |  | .03 |  | .05 |  |
|  | [-.08, .04] | | [-.09, .03] | | [-.07, .03] | | [-.02, .09] | | [-.03, .09] | | [-.07, .17] | |
| Fail to do something for dog | .13 | *** | .10 | ** | .08 | ** | -.06 |  | -.13 | *** | -.01 |  |
|  | [0.07, .19] | | [0.04, .16] | | [0.02, .15] | | [-.12, .01] | | [-.19, -.07] | | [-.11, .08] | |
| Dog greeting at the door | .01 |  | .03 |  | -.01 |  | -.03 |  | .01 |  | -.00 |  |
|  | [-.05, .08] | | [-.03, .10] | | [-.08, .05] | | [-.10, .04] | | [-.06, .08] | | [-.15, .14] | |
| Dog presence | -.01 |  | .00 |  | .02 |  | -.02 |  | .00 |  | -.05 |  |
|  | [-.06, .04] | | [-.07, .07] | | [-.03, .07] | | [-.07, .03] | | [-.05, .06] | | [-.14, .04] | |
| Friendly conversation with others due to dog presence | -.01 |  | .04 |  | -.04 |  | .07 | * | .02 |  | .08 |  |
|  | [-.07, .05] | | [-.02, .10] | | [-.10, .02] | | [0.01, .14] | | [-.04, .08] | | [-.02, .19] | |
| Problematic interaction with others while out with dog | .05 |  | .03 |  | .03 |  | .02 |  | -.02 |  | -.01 |  |
|  | [-.01, .11] | | [-.03, .10] | | [-.03, .09] | | [-.05, .08] | | [-.08, .04] | | [-.09, .06] | |
| Dog to human touching | .01 |  | .00 |  | .02 |  | .04 |  | .02 |  | .00 |  |
|  | [-.05, .07] | | [-.05, .06] | | [-.04, .08] | | [-.02, .10] | | [-.03, .08] | | [-.12, .13] | |
| Human to dog touching | .05 |  | .05 |  | .04 |  | -.01 |  | -.04 |  | -.02 |  |
|  | [-.00, .11] | | [-.01, .11] | | [-.03, .11] | | [-.07, .06] | | [-.10, .02] | | [-.13, .10] | |
| Aggressive dog behaviour | .06 |  | .05 |  | .01 |  | -.02 |  | .01 |  | -.00 |  |
|  | [-.00, .13] | | [-.02, .11] | | [-.07, .09] | | [-.09, .05] | | [-.05, .07] | | [-.12, .12] | |
| Fearful dog behaviour | .11 | ** | .11 | ** | .07 | * | -.11 | *** | -.07 | * | .03 |  |
|  | [0.04, .18] | | [0.03, .18] | | [0.00, .13] | | [-.18, -.05] | | [-.14, -.01] | | [-.09, .14] | |
| Distinct barking episodes | .03 |  | .05 |  | .01 |  | .00 |  | -.02 |  | -.00 |  |
|  | [-.03, .09] | | [-.02, .11] | | [-.06, .08] | | [-.07, .07] | | [-.08, .04] | | [-.13, .13] | |
| Destroy/chew/steal | .08 | ** | .11 | ** | .01 |  | -.06 |  | -.03 |  | -.02 |  |
|  | [0.03, .14] | | [0.04, .18] | | [-.06, .08] | | [-.12, .00] | | [-.10, .04] | | [-.13, .10] | |
| Lack of control over dog | .14 | *** | .16 | *** | .07 |  | -.11 | ** | -.09 | ** | .03 |  |
|  | [0.07, .21] | | [0.08, .24] | | [-.00, .14] | | [-.18, -.03] | | [-.16, -.02] | | [-.09, .16] | |
| House soiling | .06 |  | .07 |  | .02 |  | -.04 |  | -.04 |  | -.03 |  |
|  | [-.02, .15] | | [-.02, .16] | | [-.05, .09] | | [-.11, .03] | | [-.12, .04] | | [-.13, .06] | |
| Training dog | -.04 |  | -.05 |  | -.03 |  | .00 |  | .02 |  | .01 |  |
|  | [-.11, .02] | | [-.12, .02] | | [-.09, .02] | | [-.06, .06] | | [-.05, .08] | | [-.11, .14] | |

Note. Values are Pearson correlation coefficients and 95% confidence intervals.

*<.05, **<.01, ***<.001; p<0.001 = significance threshold for interpretation

**Table S5**. Cross-lagged coefficients for the impact of dog-related activities on owners' well-being from one week to another

| **Dog-related activity** | **Depression** | | **Anxiety** | | **Loneliness** | | **Eudaimonic well-being** | | **Hedonic well-being** | | **Suicidal ideation** | |
| --- | --- | --- | --- | --- | --- | --- | --- | --- | --- | --- | --- | --- |
| Physical health of dog | -.03 (.02) |  | -.01 (.02) |  | -.04 (.02) | * | .01 (.02) |  | .02 (.02) |  | .01 (.01) |  |
| Running/jogging with dog | .04 (.02) |  | .00 (.02) |  | .03 (.02) |  | -.03 (.02) |  | -.03 (.02) |  | -.01 (.02) |  |
| Walking dog | .05 (.02) | * | .01 (.03) |  | .01 (.02) |  | -.04 (.02) |  | -.01 (.02) |  | .03 (.02) |  |
| Fail to do something for dog | .01 (.01) |  | -.01 (.01) |  | -.00 (.01) |  | .01 (.01) |  | .01 (.01) |  | -.00 (.01) |  |
| Dog greeting at the door | .02 (.02) |  | .02 (.02) |  | .01 (.02) |  | -.03 (.02) |  | -.01 (.02) |  | .00 (.02) |  |
| Dog presence | -.01 (.04) |  | -.00 (.04) |  | .04 (.04) |  | .02 (.03) |  | -.01 (.03) |  | -.03 (.02) |  |
| Friendly conversation with others due to dog presence | .01 (.02) |  | .00 (.02) |  | -.00 (.02) |  | .02 (.02) |  | .00 (.02) |  | -.00 (.01) |  |
| Problematic interaction with others while out with dog | .01 (.01) |  | .00 (.02) |  | -.02 (.01) |  | -.00 (.01) |  | .01 (.01) |  | -.00 (.01) |  |
| Dog to human touching | .02 (.02) |  | -.01 (.03) |  | .03 (.03) |  | -.00 (.02) |  | -.00 (.02) |  | .00 (.02) |  |
| Human to dog touching | -.01 (.03) |  | -.01 (.03) |  | .01 (.03) |  | .01 (.02) |  | .03 (.02) |  | -.01 (.02) |  |
| Aggressive dog behaviour | -.01 (.02) |  | .00 (.02) |  | -.05 (.02) | * | .01 (.02) |  | .02 (.02) |  | -.00 (.02) |  |
| Fearful dog behaviour | .01 (.02) |  | .00 (.02) |  | .00 (.02) |  | -.03 (.02) | * | -.01 (.02) |  | -.00 (.01) |  |
| Distinct barking episodes | .00 (.02) |  | .00 (.02) |  | .02 (.02) |  | .00 (.02) |  | -.01 (.01) |  | -.00 (.01) |  |
| Destroy/chew/steal | .03 (.02) |  | .06 (.03) | * | -.00 (.03) |  | -.01 (.02) |  | -.02 (.02) |  | -.01 (.02) |  |
| Lack of control over dog | .03 (.02) |  | .03 (.02) |  | .00 (.02) |  | -.02 (.02) |  | -.01 (.02) |  | .00 (.02) |  |
| House soiling | -.02 (.03) |  | .01 (.04) |  | -.02 (.04) |  | -.00 (.03) |  | .01 (.03) |  | -.01 (.02) |  |
| Training dog | -.01 (.02) |  | -.01 (.02) |  | -.00 (.02) |  | -.03 (.01) | * | -.01 (.02) |  | .00 (.02) |  |

Note. Coefficients represent the number of SDs mood score increases given a doubling in the respective activity; coefficients’ standard errors in brackets.

*<.05, **<.01, ***<.001; p<0.001 = significance threshold for interpretation

**Table S6**. Cross-lagged coefficients for the impact of owners' well-being on dog-related activities from one week to another

| **Dog-related activity** | **PHQ-9** | **GAD** | **Loneliness** | **EUD** | **HED** | **Suicide** |
| --- | --- | --- | --- | --- | --- | --- |
| Physical health of dog | -.08 (.07) | -.04 (.06) | -.03 (.06) | .12 (.08) | -.03 (.07) | .00 (.21) |
| Running/jogging with dog | .12 (.06) | -.10 (.05) | .04 (.04) | .02 (.06) | -.07 (.06) | .05 (.19) |
| Walking dog | .05 (.05) | -.04 (.05) | .03 (.04) | .03 (.05) | .04 (.06) | .05 (.21) |
| Fail to do something for dog | -.04 (.09) | .11 (.08) | .02 (.07) | -.03 (.09) | .02 (.09) | -.01 (.24) |
| Dog greeting at the door | .00 (.06) | -.07 (.05) | -.01 (.05) | -.09 (.06) | -.01 (.06) | -.14 (.24) |
| Dog presence | -.03 (.04) | .04 (.03) | .03 (.03) | .01 (.03) | .03 (.03) | -.07 (.08) |
| Friendly conversation with others due to dog presence | .01 (.08) | .04 (.07) | -.09 (.06) | .04 (.08) | -.08 (.07) | .35 (.25) |
| Problematic interaction with others while out with dog | .06 (.08) | .05 (.07) | -.00 (.06) | .12 (.08) | .08 (.08) | .07 (.19) |
| Dog to human touching | -.02 (.05) | -.03 (.05) | .10 (.04) | .02 (.05) | -.07 (.05) | -.05 (.21) |
| Human to dog touching | -.05 (.06) | .06 (.06) | .11 (.05) | -.05 (.05) | .01 (.05) | -.17 (.20) |
| Aggressive dog behaviour | .03 (.08) | .10 (.07) | -.07 (.06) | -.12 (.08) | .09 (.07) | .03 (.23) |
| Fearful dog behaviour | -.02 (.08) | .09 (.07) | .01 (.06) | -.11 (.07) | .02 (.08) | -.17 (.24) |
| Distinct barking episodes | .05 (.08) | .10 (.07) | -.01 (.06) | .00 (.08) | .00 (.08) | -.10 (.28) |
| Destroy/chew/steal | .00 (.05) | .02 (.05) | .05 (.04) | -.06 (.04) | .03 (.05) | .09 (.17) |
| Lack of control over dog | .03 (.07) | .06 (.07) | -.02 (.06) | -.16 (.08) | -.03 (.07) | .13 (.23) |
| House soiling | -.05 (.04) | .05 (.04) | -.03 (.03) | -.03 (.04) | -.00 (.04) | -.03 (.10) |
| Training dog | -.06 (.07) | -.06 (.07) | .04 (.06) | -.15 (.07) | -.05 (.08) | -.10 (.29) |

Note. *<.05, **<.01, ***<.001; p<0.001 = significance threshold for interpretation
